# History of Atmospheric Cosmic Ray Research at the National Bureau of Standards

**DOI:** 10.6028/jres.125.001

**Published:** 2020-01-16

**Authors:** Bert M. Coursey

**Affiliations:** 1 National Institute of Standards and Technology, Gaithersburg, MD 20899 USA

**Keywords:** cosmic radiation, galactic cosmic rays, Geiger-Müller counters, ionizing radiation, radiometerographs, radiosondes

## Abstract

In the late 1930s, a team of physicists from the National Bureau of Standards (now the National Institute of Standards and Technology) published eight papers on the investigation of cosmic rays in the atmosphere. Payloads launched with weather balloons, also known as radiosondes, were equipped with sensors to measure temperature, relative humidity, pressure, and radiation dose. A battery-operated telemetry system was used to continuously transmit at 60 MHz to a base station. They measured the radiation dose profiles of cosmic radiation in the atmosphere up to 21 km. Calibration of the Geiger-Müller counters with a standard radium source allowed them to calculate a radiation dose rate at an altitude corresponding to 10 kPa that was 180 times the dose rate near sea level in Washington, DC. Ascents in Washington, DC (latitude 39 degrees) and Lima, Peru (near equator) allowed them to demonstrate the effects of Earth’s magnetic field on incident galactic cosmic rays; the dose rate in Peru was only half that in Washington, DC.

## 1 Introduction

From 1935 to 1939, two young physicists at the National Bureau of Standards (NBS) published eight papers on their experimental investigation of cosmic rays using weather balloons launched from the roof of buildings on the NBS campus at Connecticut Avenue and Van Ness Street in Washington, DC [1–8]. The senior of the two was 40 year old Leon Curtiss. His principal coauthor was 31 year old Allen V. Astin. They were joined by 29 year old Serge Korff from the Carnegie Institution (located 5 km south of the bureau site).

Interest in cosmic rays continues today, over a century after they were discovered in 1912. This account will focus on the state of cosmic ray research in the 1930s, and the critical contributions of the NBS team over these 4 years. By many accounts, the 1930s were considered the high point in cosmic-ray physics.

Leon Francis Curtiss received his Ph.D. in physics from Cornell University in 1922 and then spent 4 years with Ernest Rutherford at the Cavendish Laboratory in Cambridge before taking a position in the Optics Division at NBS in 1926. His experimental papers with Rutherford dealt with nuclear decay parameters for radium daughter products. Allen Varley Astin received his Ph.D. in physics from New York University in 1928, and, after postdoctoral work at Johns Hopkins University and at NBS, he took a position in the Heat and Power Division at NBS. Serge Alexander Korff obtained his Ph.D. from Princeton in 1929, and, following postdoctoral work with R.A. Millikan at the California Institute of Technology, he joined the research staff in the Department of Terrestrial Magnetism of the Carnegie Institution.

## 2 Cosmic Ray Science in the 1930s

Early radiation detectors used by Marie Curie, Ernest Rutherford, and their contemporaries relied on the fact that *ionizing radiations* caused a discharge in charged electroscopes. The currents measured with radium-226 and other radioactive sources were very low (of the order of picoamps), and reproducible measurements were difficult. The ionizing strengths of sources were compared against that of a known mass (in milligrams) of radium-226. A decade into such studies, it became clear that there was a residual background with such detectors that could not be eliminated with shielding. Victor Hess in Austria in 1912 showed that the residual background was due to a relatively constant source of ionizing radiation from the atmosphere [9]. The background exposure rate at sea level varies with location; using the old units of measure,^1^1 Throughout the paper, quantities and units are given as they were cited in the original literature. Altitudes for balloon flights are given in millibars of pressure as well as height in feet or kilometers. There are inconsistences in reported altitudes, probably due to differences in the barometric measurements and the lookup tables used by the different investigators. it can be taken as 6 µR/h (microroentgens per hour, where 1 R = 2.58 × 10^−4^ C/kg), of which about 50% is due to cosmic rays. In SI units, taking into account the biological response from high-energy particles and neutrons, this sea-level cosmic-ray dose rate is nominally 0.030 µSv/h (microsieverts per hour).

It was soon found from manned balloon flights and with aircraft-mounted detectors that the dose rates were orders of magnitude greater at high altitudes. Life on Earth is protected from the intense cosmic rays incident on Earth in two ways: The atmosphere attenuates the incident particles, and Earth’s magnetic field deflects the charged particles away from the surface. The galactic cosmic rays (GCRs) are isotropic except at very high energies. The solar system is located in the Orion Arm of the Milky Way Galaxy in the outer half of the galactic disk. The flux of GCRs (from supernovas and other cosmic sources) has no particular direction. These GCRs are 90% protons (hydrogen is the most abundant element in the universe) with about 9% doubly charged helium ions (also called alpha particles). The remaining ~1% are heavier ions. The Sun also makes episodic contributions to the energetic particle flux at the top of the atmosphere (solar cosmic radiation). This contribution is more strongly dominated by protons. These solar energetic particles are accelerated by explosive energy releases by the Sun (solar flares and coronal mass ejections). The frequency of these outbursts varies with Sun spot activity. These particles are mostly at lower energies than the GCRs, and hence they are strongly attenuated by Earth’s atmosphere.

We do not know the origins of the most energetic GCRs, up to 10^20^ electron volts (eV).^2^2 The Large Hadron Collider at the Conseil Européen pour la Recherche Nucléaire (CERN), the European Organization for Nuclear Research, produced particles up to 14 TeV (14 × 10^15^ eV) in the recent search for the Higgs Boson particle. When, after millions of years in space, these GCRs strike atmospheric matter, they create secondary hadrons and mesons (primarily pions or π-mesons), which decay in less than nanoseconds. These interactions result in showers of lower energy particles. The hadronic component of these showers becomes mostly neutrons as the showers proceed down into the atmosphere. The pion component consists of charged pions (π^+^ and π^−^) that rapidly decay into muons. Neutral pions are also produced (π^0^). Each π^0^ very quickly decays into a pair of gamma rays. The gamma rays produce electron-positron pairs, and these electrons go on to generate bremsstrahlung radiation, thereby producing more gamma rays. This process develops an electromagnetic component to the shower, which is typically the dominant source of the ionizing radiation through much of the atmosphere.

The nature of the shower particles was predicted by Carlson and Oppenheimer in 1937 [10]. Pions were predicted in 1935 but were not detected until 1947. Carl Anderson discovered the positron in cosmic rays in 1932 [11] and, with Seth Neddermeyer, the muon in 1936 [12].

## 3 Radiosonde Development in the United States

In the first paper from Curtiss and Astin, published in November 1935 in the *Journal of Aeronautical Sciences* [1], they described a practical system of radiometerography. This was essentially an instrument package that could be sent aloft in an unmanned weather balloon and transmit data of meteorological interest to a ground receiver. They were not the first. These systems had been under intensive development worldwide for over a decade. A Frenchman, Robert Bureau, in 1929 gave them the name “radiosondes” [13]. The essential components of a radiosonde were meteorographic instruments to measure pressure, temperature (T), and relative humidity (RH), a clock (or constant-speed electrical motor), a radio transmitter, batteries, and a ground receiver. A mechanical system was needed to key the transmitter to signals from the meteorological instruments. Curtiss and Astin used a modification of the earliest telemeteorograph designed by H. Olland in The Netherlands in 1877 [14]. Their system, which weighed less than 1 kg, transmitted data to altitudes up to 23 km. They used a 60 MHz transmitter. A wavelength of 5 m was used, permitting use of half-wave tuned doublet antennas for transmission and reception. It is worth noting that they did not actually measure “altitude.” They measured pressure at sea level and during ascent (and descent) of the balloon with an aneroid barometer. The altitude was calculated from the classic equations of barometric hypsometry. The atmosphere is considered an ideal gas, and its mass is subject to the gravitational force of Earth. Corrections are required for temperature and relative humidity [15]. The pressure sensor was a “small aneroid capsule of copper-beryllium alloy.[8]”

They did not indicate in this paper the instruments used for T and RH. However, the history of the radiosonde, prepared by Dubois, Multhauf, and Zeigler at the Smithsonian Institution in 2002, reports that the NBS design initially (1936) used a hair hygrometer for relative humidity and an electric thermometer (liquid-filled tube with a nonfreezing electrolyte) for temperature [16]. The hair hygrometer and temperature sensor were not suitable for measurements at high altitudes, and NBS replaced them with electronic sensors in a combined unit.

**Fig. 1:**
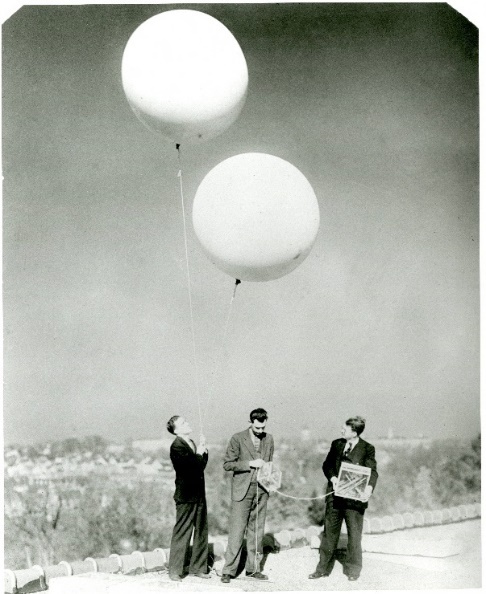
A.V. Astin, L.L. Stockman, and L.F. Curtiss with radiosonde ready to launch in 1936. Photograph from National Institute of Standards and Technology (NIST) archives.

**Fig. 2:**
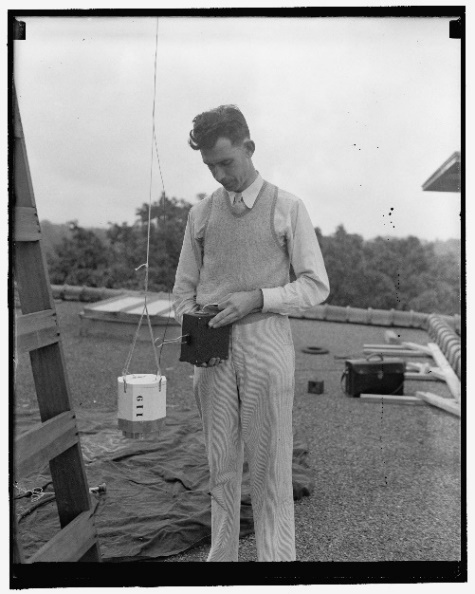
L.L. Stockman attaching radiosonde to balloon circa 1936. Photograph from NIST archives.

**Fig. 3:**
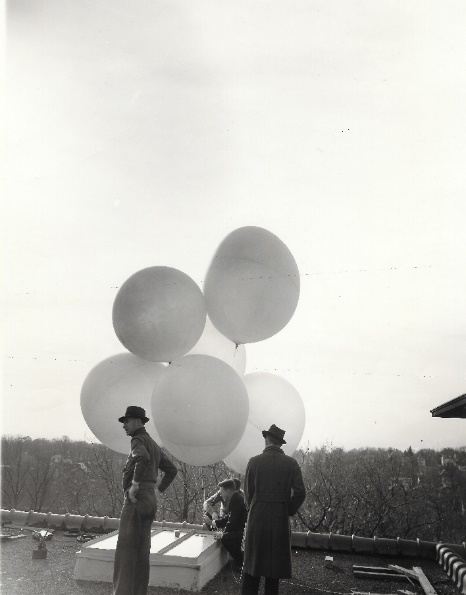
NBS team preparing another radiosonde launch from the roof of a building at NBS. Multiple balloons worked better because they would not all burst at the same altitude, which reduced the velocity of the package on descent. Photograph from NIST archives.

The 1935 paper acknowledges assistance from two other young NBS collaborators, Leroy Stockman and Burrell Brown, who worked with Curtiss in the Atomic Physics Group in the Optics Division. In the same year, Astin and Stockman published a paper in the *Review of Scientific Instruments* on the receiver used for the radiometeorographs [2]. The receiver was to have sufficient sensitivity to allow recording at distances of 80 to 160 km from the release point and have sufficiently broad tuning that it did not have to be retuned during flights. This paper, with Astin as the first author, is the best indication that he was brought on to assist the Atomic Physics Group because of his expertise in electronics. Figures 1, 2, and 3 show the NBS team preparing the balloons for launch on the roof of a building at the NBS campus in Washington, DC.

The 1935 papers express appreciation for discussions with the U.S. Weather Bureau in Washington, DC, and the Blue Hill Meteorological Observatory in Milton, Massachusetts. The Weather Bureau assisted with balloon technology. The Blue Hill team had decades of experience in weather research and were also developing radiosondes. They also cite the Bartol Foundation. W.F.G. Swann of the Bartol Research Foundation (now a part of the Department of Physics at the University of Delaware) was also engaged in balloon studies of cosmic rays with assistance from the Army Air Corps [17]. They did not mention support from the U.S. Navy on this research. However, according to the Smithsonian history [16], in 1936, the Navy asked NBS to develop a radiosonde. “Harry Diamond and his associates at NBS organized a well-financed program that produced a superior radiosonde” [18].

This suggests an interesting divergence of efforts as Curtiss and his team in the Atomic Physics Group began a collaboration with Serge Korff to study cosmic rays, and Diamond’s team in the Radio Group of the Electricity Division focused on the applications of telemetry for military aviation (wind speed and direction become important, as well as temperature [T], pressure [P], and relative humidity [RH]). Radiosondes developed by both teams used similar instrumentation packages, but Diamond introduced the multichannel capability in the telemetry that allowed the increase in bandwidth for the high volume of data that came from the “NBS-Navy” radiosonde. The subcarrier audio-frequency could be modulated as a means of relaying T, P, and RH. This radiosonde met the Navy’s requirements that it cost less than $25 and weighed less than 1 kg. It was quickly mass produced by commercial firms and was available for U.S. military applications in World War II. The NBS director, Dr. Lyman Briggs, was doubtless pleased with these research efforts, which contributed to both basic science and the agency missions of the Weather Bureau and the Department of Defense [19].

Both groups published in the NBS *Journal of Research*, but there are no joint publications by Curtiss, Astin, and Diamond. Curtiss and Astin did get the priority publications for the physics community. Their 1936 note in *Science* [4] reports a March 1936 balloon ascent to a minimum atmospheric pressure of 8 mbar, corresponding to an altitude of 38.7 km (127,000 feet). The balloon had an ascension rate of 500 m/min, carrying a 5 m transmitter and associated equipment that weighed less than 1 kg. Newspapers had reported in April that a Russian team had made a balloon ascent to a pressure corresponding to 139,000 feet [20]. This seems to be the first of several space races with the Russians over the next eight decades. In the 1938 *Physical Review* paper [6], Curtiss *et al*. amended their earlier claim on altitude; they recorded data to a pressure of 5 mbar, corresponding to an altitude of 116,000 feet. This correction was necessary because in the earlier paper they had used an “incorrect altitude table.”

## 4 NBS Radiosondes and Cosmic-Ray Measurements

The year 1938 saw Curtiss and Astin’s first publication in *Physical Review* on cosmic-ray measurements, with Serge Korff as first author [5]. Millikan and collaborators had previously reported on electroscope measurements made up to 8.8 km (29,000 feet) in Peru [21]. Korff was the explorer who was able to finance expeditions around the world to measure the influence of Earth’s magnetic field on the cosmic-ray flux. Curtiss and Astin were able to provide lower weight, ruggedized, and higher accuracy meteorological devices and new custom-designed Geiger-Müller (G-M) counters to measure cosmic rays. Curtiss, following the legacy of his 4 years at the Cavendish Laboratory with Rutherford and Chadwick, was an expert in radiation detector design and construction. He had already developed the U.S. national standard gold-leaf electroscope [22], and later published an NBS Circular on Geiger-Müller counters [23]. They set out the criteria for counter equipment for balloon work as: (a) light weight, (b) strong pulse with minimum number of tubes, (c) a sea-level counting rate such that the vastly increased counting rate at highest altitudes would still be within the response time constant of the relay or whatever other device was the slowest in the circuit, (d) ruggedness, and (e) proper operation under conditions of varying temperature and pressure. With these considerations in mind, they built a number of G-M counters, varying the filling gas (helium or argon), volume, gas pressure, and operating voltage. After measurement of a sea-level count rate of less than 1 count per minute (1 cpm), the counter was exposed to a radium gamma-ray source to ensure that it would operate satisfactorily at up to 200 cpm.

**Fig. 4:**
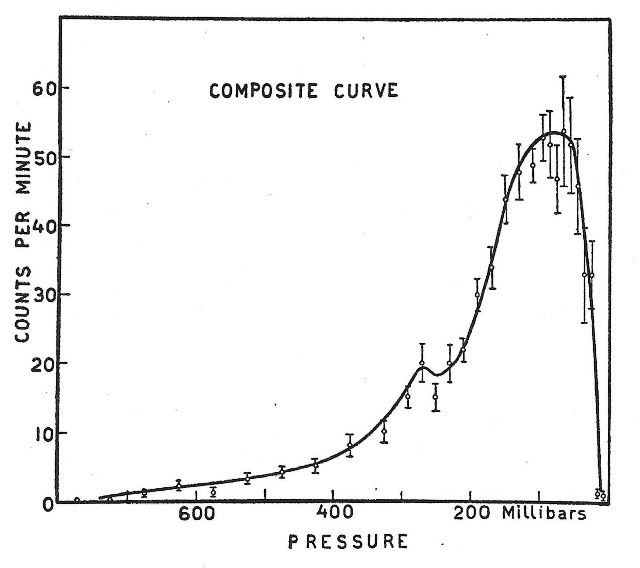
From Curtiss *et al.*, *Physical Review*, 1938 [6], showing a composite curve from average value of cosmic-ray counts obtained from the 10 highest flights. Vertical lines represent the probable error of the arithmetic mean.

Measurements of the atmospheric profile of cosmic rays were carried out in Washington, DC (latitude 39 degrees north) at altitudes up to 70,000 feet. Typical results are shown in Fig. 4. The cosmic-ray intensity as measured by the G-M counting rate showed a rapid increase at pressures below 400 mbar (40 kPa) and reached a maximum at about 100 mbar (10 kPa). Measurements were made up to the top of the atmosphere (about 8 mbar) until the balloons burst. The counting rate measurements during the descents were also collected and were consistent in terms of pressure versus counting rate.^3^3 Current National Weather Service radiosondes are expected to reach 40 kPa (400 mbar), corresponding to 7.0 km (23,000 feet), in order to transmit useful weather data. This maximum counting rate at 100 mbar at the Washington, DC, latitude corresponds to approximately 15.24 km (50,000 feet).

With balloon ascents that Korff was able to make in Lima, Peru (magnetic latitude 0 degrees), they established that the shape of the cosmic-ray profile was similar, but the intensity of the counting rate in Lima at 45,000 feet was only 50% of that in Washington, DC. The altitude corresponding to the maximum rate was lower in Peru. They attributed the difference as “due to the fact that the softer part of the incoming radiation is cut off by the Earth’s magnetic field and does not reach the top of the atmosphere at the equator. Those rays that produce ionization in the upper atmosphere in the lower latitudes are therefore harder and penetrate further into the atmosphere before they come into equilibrium with their secondaries [5].”

They reported agreement with Millikan’s results, which showed that counting rate decreased by 20 to 25% from the maximum as the balloons approached the top of the atmosphere. Millikan first interpreted this to suggest that the incident cosmic rays at the highest altitudes were not ionizing; that is, they were uncharged photons. In their discussion [5], Korff *et al.* added support to Millikan, stating that neutrons or photons could constitute the cosmic rays at these altitudes. It was later realized that these positively charged hadrons (mainly protons) have a dearth of targets as they enter the atmosphere. This explains the increase in counting rate as secondary electrons are produced with increasing density of molecular nitrogen and oxygen.

## 5 Curtiss, Astin, and Cosmic-Ray Metrology

In their last three papers on cosmic rays and radiometeorography, Curtiss and Astin refined and consolidated their experimental techniques, validated their measurements, and reported uncertainties for the meteorographic and radiologic parameters. Leroy Stockman and Burrell Brown were coauthors. The second 1938 *Physical Review* paper (Curtiss was the first author [6]) reported on averaging of results from G-M measurements of cosmic rays for 18 successful balloon ascensions over a 6-month period in 1937. All of these balloon experiments were done in Washington, DC, so the cosmic-ray air-ionization profiles were limited to the 39 degrees latitude. By averaging over many flights, they were able to identify a secondary maximum of intensity at about 30 kPa (300 mbar). (The maximum was at 10 kPa or 100 millibars.) This secondary peak was not identified in the measurements in Peru, so the authors suggested that it was due to the shape of Earth’s magnetic field and would be more pronounced at higher latitudes. More definitive papers on the latitude effect were obtained by other investigators who mapped the cosmic-ray exposure on flights from the equator to northern latitudes.

The last two papers that Curtiss and Astin published on cosmic rays (with Stockman and Brown as coauthors) were in the NBS *Journal of Research*. This allowed them to give far more details on the design of each piece of equipment in the radiosonde as well as detailed statistics for the cosmic-ray measurements. They also reported on a new design for the G-M counter. They replaced the glass tube used in the earlier work with a copper-tube cathode with a steel-wire anode. This resulted in a thirtyfold increase in counting rate, which greatly improved counting statistics. They standardized each counter by measuring the counting rate at 1 m from a 1 mg radium-226 standard source. The standard counting rate was about 3000 counts per minute. This may have been the first attempt to actually measure the dose rate from cosmic rays at high altitudes. They made 15 balloon ascents in 1938, which allowed them to establish the reproducibility of the results. The highest altitudes attained were to pressures of 1 kPa (10 mbar) (100,000 ± 5000 feet). In these experiments, they found a maximum counting rate at 58 ± 1 mbar, corresponding to 69,500 feet. The maximum counting rate at 58 mbar was 55% of the rate measured with the radium standard. They did not report a “standard exposure rate” for the radium-226. Such precision dosimetry measurements were in their infancy at NBS at the time by their coworker Lauriston Taylor. However, a nominal value for their standard would be 0.84 Roentgen per hour per gram at 1 m. This corresponds to 840 µR/h per milligram at 1 m. So, their maximum cosmic-ray exposure rate (relative to radium gamma rays) would be of the order 460 µR/h. This would give a rate 150 times that at sea level. Their own estimates derived from counting rates at sea level and at altitudes was a factor of 180. Of course, these should not be interpreted as actual dose rates, because the fluxes of charged particles and neutrons at these altitudes begin to dominate the ambient dose equivalent in sieverts [24,25].

**Fig. 5:**
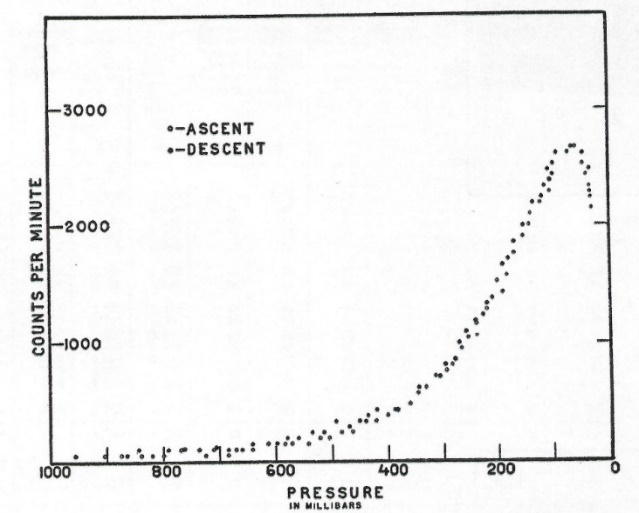
From Curtiss *et al.* NBS Journal of Research, 1939 [8]. Data obtained on a single ascent and descent, showing counts per minute plotted against the atmospheric pressure in millibars.

They did not explain two apparent discrepancies with their 1938 *Physical Review* paper. (1) Why did their maximum counting rate occur at 58 mbar rather than 100 mbar as reported previously? (2) Why did they not observe a secondary maximum in the later ascents, which had much better statistics? The cosmic-ray profiles in the two sets of experiments are shown in Figs. 4 and 5. The discrepancy in the altitude corresponding to maximum counting rate may be due to a difference in the geometry and material construction of the G-M tubes. The counters respond to secondary electrons, and the thicknesses and *atomic number (Z)* of the wall materials might cause a shift in the observed maximum counting rate with respect to pressure. Also, perhaps, there could have been a difference due to the altimeters used in the experiments. The failure, in the last ascents, to observe a second peak on the shoulder is also puzzling. Since there is no clear physical basis for this second peak, it was likely a measurement artifact.

**Fig. 6:**
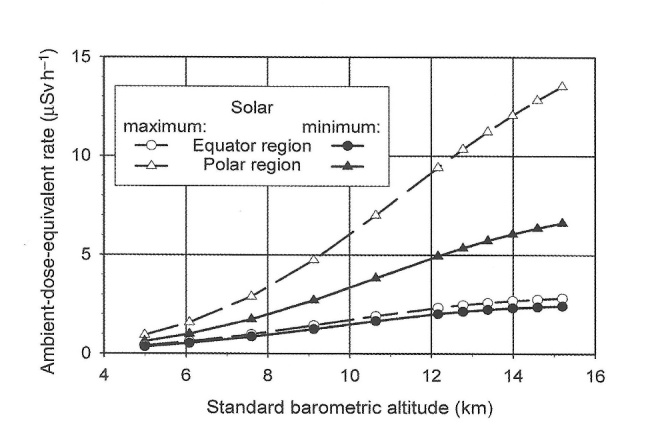
Calculated ambient-dose-equivalent rate, dH*(10)/*d*t, for conditions close to solar maximum activity (open symbols) and close to solar minimum (closed symbols), at zero meridian (*i.e.*, 0 degrees longitude) and at geographic latitudes, 0 degrees (circles) and 90 degrees (triangles) [25, 26]. From Fig. 3.1 in ICRU Report 84 [25].

It is interesting to look at Curtiss and Astin’s pioneering investigations in perspective after eight decades of cosmic-ray research. There were a number of parameters that they could not properly bound at the time, as can be shown in the following three figures from International Commission on Radiation Units and Measurements (ICRU) Report 84, which looked at cosmic-ray exposures to aircraft crews [24, 25]. Figure 6 shows how the ambient dose equivalent rate varies with altitude and the solar cycle.

**Fig. 7:**
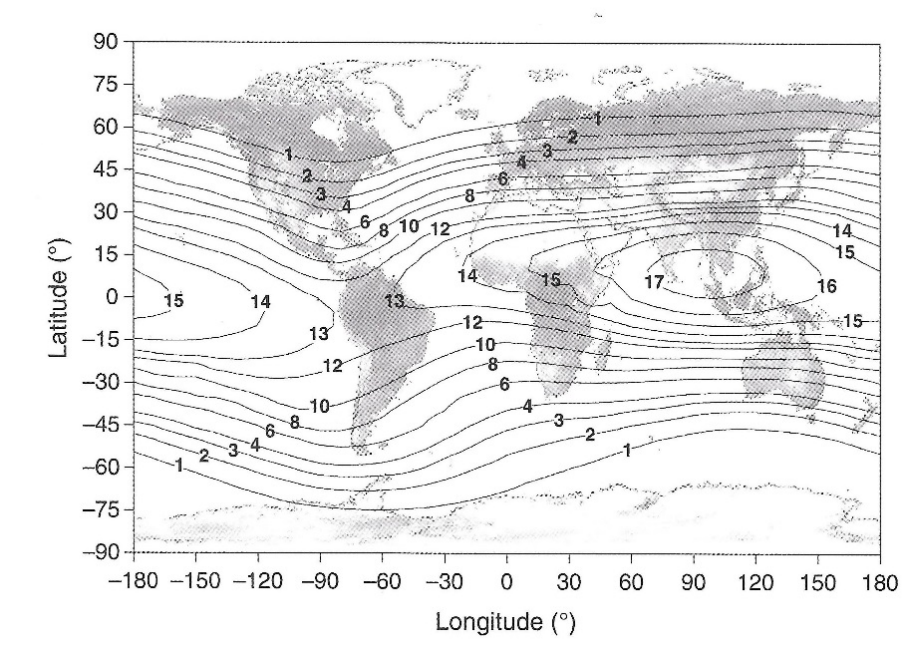
Vertical geomagnetic cutoff rigidities (GV) based on data in 1990 at a 20 km altitude. The background world map is modified, but originally taken from the “Visible Earth” catalog of the National Aeronautics and Space Administration [24, 26, 27]. From Fig. 3.2 in ICRU Report 84 [25].

**Fig. 8:**
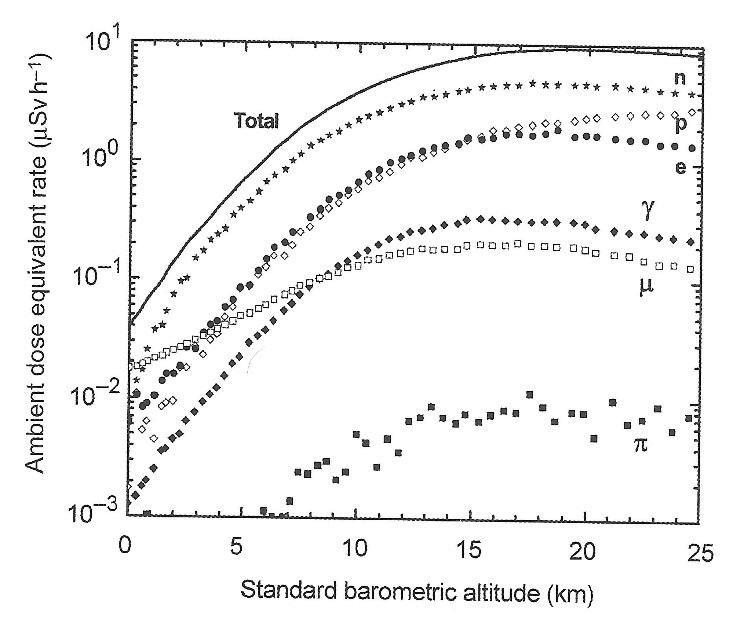
Ambient-dose-equivalent rates as a function of standard barometric altitude at 2 GV vertical geomagnetic cutoff rigidity and mid–solar cycle, for various particles of the cosmic radiation field in the atmosphere calculated using the Monte Carlo radiation-transport code FLUKA. From ICRU Report 84 (Fig. 4.2) and references therein [25].

First, since they were making balloon ascents over several years, they could not ensure that discrepancies between data sets were not due to different solar cosmic-ray conditions. Second, even though they were aware of a latitude effect, they were only sampling from two points on Earth: Washington, DC, and Lima, Peru. As Fig. 7 shows, the cosmic-ray flux over Earth’s surface is quite complex; it is generally higher at the poles and lower at the equator. The minimum over the Indian Ocean is due to the fact that Earth’s magnetic center is offset from the geometric center. Finally, Fig. 8 shows the contributions of the various particles (protons, neutrons, electrons, *etc*.) to the combined ambient dose equivalent. It has taken decades of experimental work and modeling to sort out the contributions of each particle as a function of altitude, taking into account geomagnetic conditions and solar activity. The G-M counters and electroscopes used in the initial measurements could only respond to secondaries created in the working gas of the detectors.

## 6 Summary

Curtiss and Astin chose not to venture into two parallel research efforts in the 1930s: radio engineering and particle physics. While Harry Diamond’s group at NBS worked with the U.S. Navy to develop a multifrequency transmitter, Curtiss’ team chose instead to optimize their 5 m transmitter, antennae construction, and ground receiver, which proved quite adequate for their purposes. Also, they did not explore cloud chambers and photographic emulsions to record high-energy particle tracks from cosmic rays. The Carnegie Institution, California Institute of Technology, and many university physics departments were already active in this research. By the late 1930s, the experimental data from particle-track analysis, the availability of high-altitude aircraft flights and their ability to carry multiple detectors, and the growing body of work from theorists on quantum electrodynamics were converging to allow a reasonable picture of the nature of cosmic rays arriving at the top of Earth’s atmosphere, and their behavior on penetration to Earth’s surface. Where the GCRs originated and how their progeny could be used for physics and engineering studies on Earth and in space were not yet realized.

What Curtiss and Astin and their collaborators were able to do was make valuable contributions to the development of inexpensive, robust, and accurate radiosondes for weather research. Present-day radiosondes used by the National Weather Service differ very little from those developed at NBS. They also contributed valuable information at the time to the understanding of dose rate profiles and the latitude effect for cosmic rays from the edge of the atmosphere to the surface. Their last paper in 1939, in the NBS *Journal of Research*, was perhaps the first report of measured radiation dose rate from cosmic rays [8]. Radiation doses to travelers at high altitudes was not understood as a problem in 1939. Today, however, accurate calculations and measurements of radiation doses are essential to understanding potential health effect for airline crews and astronauts who spend a great deal of their time at high altitudes without the full benefits of shielding by the atmosphere and Earth’s magnetic field [24, 25].

## 7 Epilogue

The collaboration of these young physicists propelled each of them to leadership in U.S. science in the mid-twentieth century. Leon Curtiss became a section chief at the NBS, first for radioactivity and then for neutron physics. He was named chair of a National Research Council panel on nuclear science. Serge Korff became a professor of physics at New York University. He was a president of the Explorer’s Club of New York and also a president of the American Geographical Society. Allen Astin moved to the Electricity Division and worked on the proximity fuse during World War II. In 1945, Astin was the assistant to Harry Diamond in the NBS Ordnance Department. He was quickly recognized for his administrative and leadership capabilities and in 1951 was selected as the fifth director of NBS. Astin is best remembered by American scientists for his principled stand against junk science, the AD-X2 battery acid controversy. In later years, Astin created a position for his old colleague Curtiss as “Consultant to the Director.”
